# Identification of a PD-L1^+^Tim-1^+^ iNKT subset that protects against fine particulate matter–induced airway inflammation

**DOI:** 10.1172/jci.insight.164157

**Published:** 2022-12-08

**Authors:** Christina Li-Ping Thio, Alan Chuan-Ying Lai, Jo-Chiao Wang, Po-Yu Chi, Ya-Lin Chang, Yu-Tse Ting, Shih-Yu Chen, Ya-Jen Chang

**Affiliations:** 1 Institute of Biomedical Sciences, Academia Sinica, Taipei, Taiwan.; 2 Institute of Translational Medicine and New Drug Development, China Medical University, Taichung, Taiwan.

**Keywords:** Immunology, Inflammation, Asthma, Innate immunity, NKT cells

## Abstract

Although air pollutants such as fine particulate matter (PM_2.5_) are associated with acute and chronic lung inflammation, the etiology of PM_2.5_-induced airway inflammation remains poorly understood. Here we report that PM_2.5_ triggered airway hyperreactivity (AHR) and neutrophilic inflammation with concomitant increases in Th1 and Th17 responses and epithelial cell apoptosis. We found that γδ T cells promoted neutrophilic inflammation and AHR through IL-17A. Unexpectedly, we found that invariant natural killer T (iNKT) cells played a protective role in PM_2.5_-induced pulmonary inflammation. Specifically, PM_2.5_ activated a suppressive CD4^–^ iNKT cell subset that coexpressed Tim-1 and programmed cell death ligand 1 (PD-L1). Activation of this suppressive subset was mediated by Tim-1 recognition of phosphatidylserine on apoptotic cells. The suppressive iNKT subset inhibited γδ T cell expansion and intrinsic IL-17A production, and the inhibitory effects of iNKT cells on the cytokine-producing capacity of γδ T cells were mediated in part by PD-1/PD-L1 signaling. Taken together, our findings underscore a pathogenic role for IL-17A–producing γδ T cells in PM_2.5_-elicited inflammation and identify PD-L1^+^Tim-1^+^CD4^–^ iNKT cells as a protective subset that prevents PM_2.5_-induced AHR and neutrophilia by inhibiting γδ T cell function.

## Graphical abstract

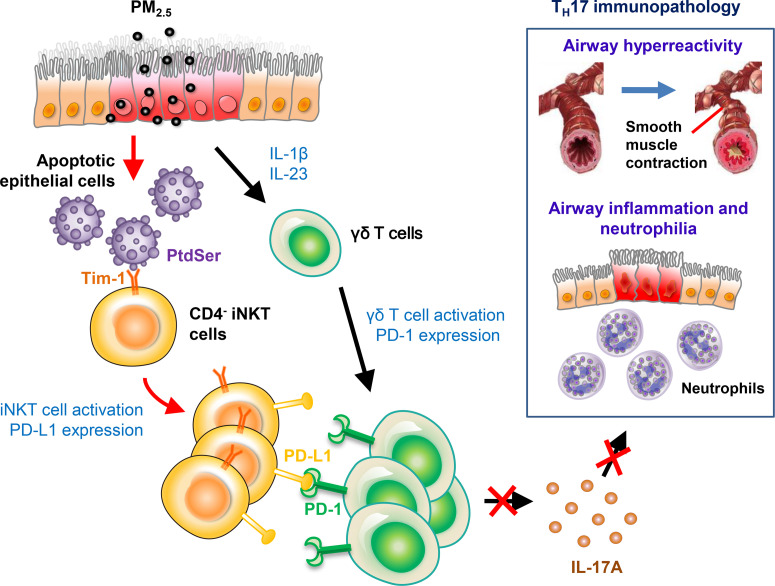

## Introduction

Air pollution is a factor associated with hospitalization for respiratory diseases and, therefore, is a cause of increased medical care burden worldwide (1–3). Toxicities and biological effects of ambient particulate matter (PM) depend on the particle diameter and source. PM with a diameter of 10 μm, known as PM_10_, is mostly restricted to the upper airway and is removed through breathing. Particles smaller than 10 μm, especially those with diameters of 2.5 μm or less, known as fine particulate matter (PM_2.5_), can reach distal airways and can accumulate (4). Clinically, short-term exposure to PM_2.5_ results in increased risk of wheezing in children with asthma (5), and long-term exposure results in the development of poorly controlled asthma and reduced lung function in both children and adults (6). Nevertheless, the pathophysiology of PM_2.5_-induced airway inflammation and asthma remains unclear.

Invariant natural killer T (iNKT) cells are a subset of T cells with a restricted TCRα chain (Vα14-Jα18 in mouse and Vα24-Jα18 in human) (7). This T cell receptor (TCR) rearrangement enables iNKT cells to recognize lipids and glycolipids presented by CD1d on antigen-presenting cells. iNKT cells are a heterogeneous population with 3 major subsets, NKT1, NKT2, and NKT17, which produce IFN-γ, IL-4, and IL-17A, respectively (8). iNKT cells can either inhibit or exacerbate allergic responses. For instance, iNKT cells activated by α-galactosylceramide (9) exert suppressive function, whereas iNKT cells activated by house dust extracts promote ovalbumin-induced sensitization (10, 11). iNKT cells play protective roles in autoimmune diseases, including multiple sclerosis and rheumatoid arthritis (12).

γδ T cells are a specialized T cell population that bear the γδ TCR instead of the conventional αβ TCR; these cells are found in various mucosal sites such as the skin and lungs (13). These T cells exhibit “innate-like” properties: they do not engage MHC antigen but can be activated directly by TLR stimulation to produce immunomodulatory regulators as the first line of defense (14). This is especially true for IL-17A–producing γδ T cells, which, unlike Th17 cells, can differentiate into Th17 lineage in response to IL-1β and IL-23 without TCR stimulation (15). IL-17A–producing γδ T cells are critical in the early defense against bacterial infection (16). Their role in asthma remains controversial, with studies showing both protective and pathogenic roles in allergen-induced asthma (17, 18).

In this study, we found that PM_2.5_ induces acute neutrophilic inflammation and airway hyperreactivity (AHR) accompanied by mixed Th1 and Th17 responses. Pathological analysis revealed that PM_2.5_ exposure induces pulmonary cell apoptosis, alveolar leakage, and epithelial cell hypertrophy. Pulmonary γδ T cells and iNKT cells are activated after PM_2.5_ exposure. Whereas γδ T cell–derived IL-17A contributes to PM_2.5_-induced lung pathogenesis, iNKT cells confer protection by suppressing γδ T cell function. Detailed analysis revealed that activation of a suppressive CD4^–^ iNKT cell subset that coexpresses Tim-1 and programmed cell death ligand 1 (PD-L1) blocks γδ T cell function in part through PD-1/PD-L1 signaling. This suppressive subset is activated by apoptotic cells through recognition of phosphatidylserine (PtdSer) by Tim-1, which is upregulated specifically on the CD4^–^ iNKT subset upon PM_2.5_ exposure. In sum, this study demonstrates the pathogenic function of γδ T cells in PM_2.5_-mediated airway inflammation and AHR and underscores the protective function of the Tim-1 and PD-L1 coexpressing CD4^–^ iNKT cell subset in air pollutant–induced lung pathogenesis.

## Results

### PM_2.5_ induces acute AHR and airway inflammation characterized by neutrophilic inflammation.

**Figure 1:**
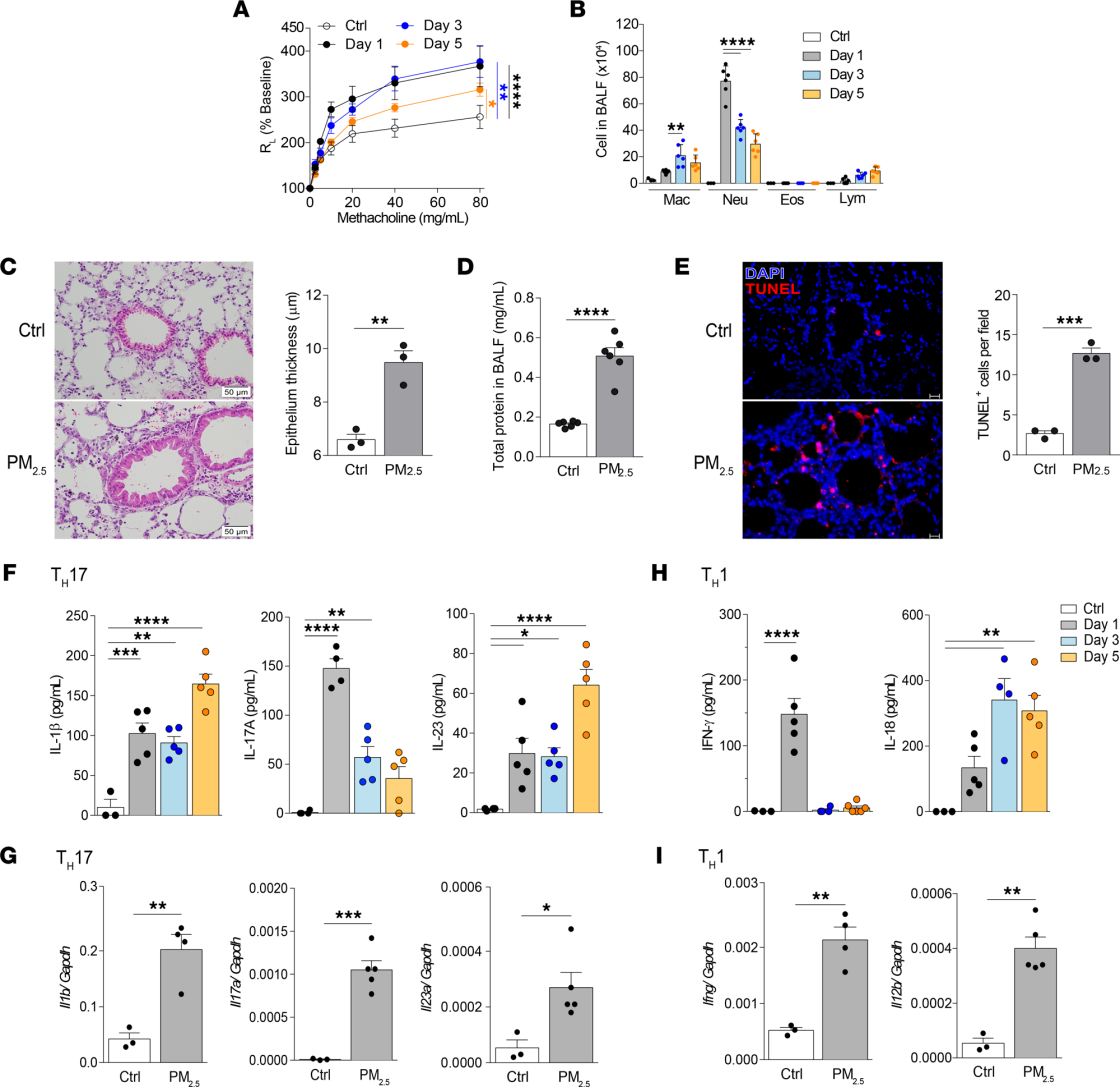
Kinetics of PM_2.5_-induced AHR and airway inflammation. (**A** and **B**) BALB/c mice (WT) received daily i.n. exposure to PM_2.5_ for 3 days and were sacrificed 1, 3, or 5 days after the last exposure. (**A**) Lung resistance in response to increasing doses of methacholine. (**B**) Cellular composition in BALF. (**C**–**I**) WT mice received daily i.n. exposure of PM_2.5_ for 3 days and were sacrificed 1 day after the last exposure. (**C**) Representative images of H&E-stained histologic sections of lung tissues and quantification of bronchial thickness. (Scale bars: 50 μm; original magnification, ×40.) (**D**) Total protein in BALF. (**E**) Immunofluorescence staining of apoptotic cells (by TUNEL assay) and quantification of TUNEL^+^ pulmonary cells. (Scale bars: 20 μm; original magnification, ×20.) (**F** and **G**) Protein (**F**) and gene expression (**G**) levels of Th17-related cytokine (IL-1β, IL-17A, and IL-23) in the BALF and lungs, respectively. (**H** and **I**) Protein (**H**) and gene expression (**I**) levels of Th1-associated cytokines (IFN-γ, IL-12b, and IL-18) in the BALF and lungs, respectively. Data are shown as mean ± SEM from 2 independent experiments (*n* = 3–6 per group) (**A**, **B**, **D**, and **F**–**I**) or representative of 2 independent experiments with consistent findings (*n* = 3 per group) (**C** and **E**). Statistical analysis was performed using 2-way ANOVA (**A**), 1-way ANOVA (**B**, **F**, and **H**), or an unpaired 2-tailed *t* test (**C**–**E**, **G**, and **I**). *P < 0.05, **P < 0.01, ***P < 0.001, and ****P < 0.0001. Ctrl, control; Eos, eosinophil; Lym, lymphocyte; Mac, macrophage; Neu, neutrophil.

We first investigated the kinetics of PM_2.5_-induced AHR and airway inflammation in mice. Mice were exposed to 200 μg of PM_2.5_ once daily for 3 days and were sacrificed 1, 3, or 5 days after the last exposure. AHR was quantified by measuring lung resistance in response to methacholine. We observed that PM_2.5_ increased airway resistance on days 1 and 3 after exposure to a similar degree, whereas resistance was markedly lower 5 days after the last exposure (Figure 1A). Neutrophil numbers in bronchoalveolar lavage fluid (BALF) were profoundly increased upon exposure to PM_2.5_, peaking on day 1 after exposure (Figure 1B). Notably, no eosinophils were detected throughout the duration of the experiment. Consistent with an acute response, H&E staining of lung tissue sections at 1 day after the last exposure revealed bronchial epithelium thickening (Figure 1C). Likewise at this time point, total protein concentration in BALF was increased 2.5-fold, indicating alveolar leakage (Figure 1D). Furthermore, pulmonary cell apoptosis increased after exposure to PM_2.5_, as detected by TUNEL assay (Figure 1E).

Next, we examined the levels of inflammatory cytokines. The levels of Th17-associated cytokines (namely, IL-17A, IL-1β, and IL-23) were elevated as early as day 1 after exposure (Figure 1F). IL-17A kinetics mirrored that of neutrophils in the BALF (Figure 1B), whereas IL-1β and IL-23 levels in the BALF increased continuously throughout the observation period. Consistent with the protein levels, mRNA levels of all 3 cytokines were increased in PM_2.5_-exposed mice (Figure 1G). The Th1-related cytokines IFN-γ and IL-18 were also induced by PM_2.5_ at both the protein (Figure 1H) and mRNA (Figure 1I) levels. Of note, PM_2.5_ did not trigger a Th2 response (Supplemental Figure 1A; supplemental material available online with this article; https://doi.org/10.1172/jci.insight.164157DS1). Further confirming the lack of a Th2 response was the observation that IL-13 deficiency did not affect the levels of BALF neutrophils, IL-17A, or neutrophil chemoattractant-encoding *cxcl1* and *cxcl2* (Supplemental Figure 1, B–D). Overall, these data indicate that PM_2.5_ induces acute airway obstruction, neutrophilic inflammation, and pulmonary cell apoptosis with concomitant Th1/Th17–biased cytokine production.

### IL-17A derived from γδ T cells contributes to PM_2.5_-mediated lung inflammation.

**Figure 2:**
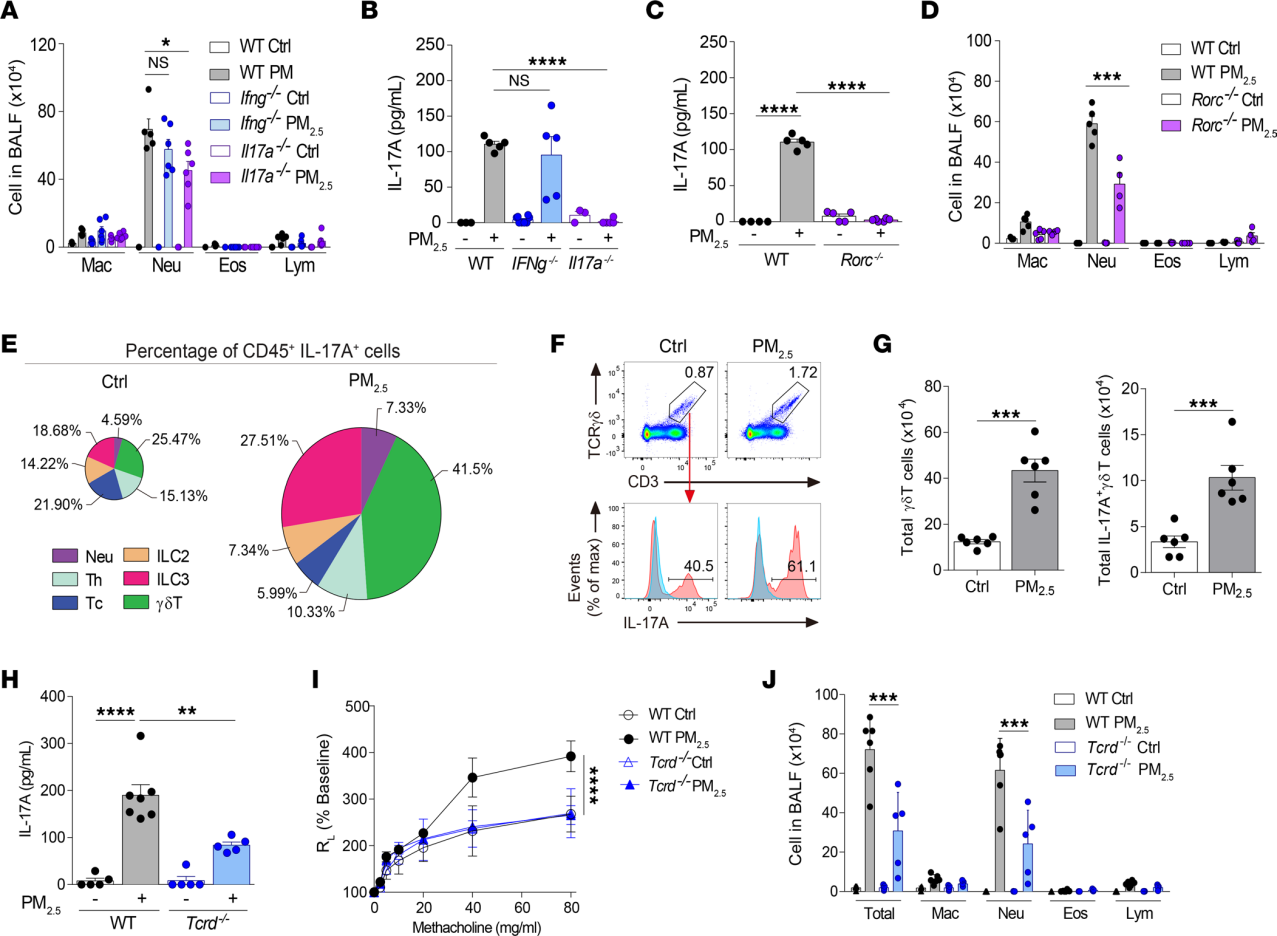
γδ T cell–derived IL-17A mediates the pathogenicity of PM_2.5_. (**A** and **B**) C57BL/6 (WT), *Ifng^–/–^*, and *Il17a^–/–^* mice received daily i.n. exposure of PM_2.5_ for 3 days and were sacrificed 1 day after the last exposure. (**A**) Cellular composition in BALF. (**B**) IL-17A level in BALF. (**C** and **D**) WT and *Rorc^–/–^* mice received daily i.n. exposure of PM_2.5_ as in **A**. (**C**) IL-17A level in BALF. (**D**) Cellular composition in BALF. (**E**–**G**) WT mice received daily i.n. exposure of PM_2.5_ as in **A**. (**E**) Percentages of IL-17A–producing CD45^+^ cells in the lungs. Cells are gated as follows: γδ T cells (CD3^+^TCRγδ^+^ cells), Th cells (CD3^+^CD4^+^ cells), cytotoxic T cells (CD3^+^CD4^–^ cells), neutrophils (Ly6G^+^ cells), ILC2 (Lin^–^GATA3^+^ cells), and ILC3 (Lin^–^RORγt^+^ cells). (**F** and **G**) Representative flow cytometry plot showing IL-17A–producing γδ T cells (**F**) and quantification of total γδ T cells and total IL-17A–producing γδ T cells (**G**). Blue solid line: Isotype-matched control; red solid line: Ab staining. (**H**–**J**) WT and *Tcrd^–/–^* mice received daily i.n. exposure of PM_2.5_ as in **A**. (**H**) IL-17A level in BALF. (**I**) Lung resistance in response to increasing doses of methacholine. (**J**) Cellular composition in BALF. Data are shown as mean ± SEM from 2 independent experiments (*n* = 4–7 per group). Statistical analysis was performed using 1-way ANOVA (**A**–**D**, **H**, and **J**), an unpaired 2-tailed *t* test (**G**), or 2-way ANOVA (**I**). *P < 0.05, **P < 0.01, ***P < 0.001, and ****P < 0.0001. Ctrl, control; Eos, eosinophil; Lym, lymphocyte; Mac, macrophage; Max, maximum; Neu, neutrophil; Tc, cytotoxic T cell.

IL-17A is associated with AHR and neutrophilic inflammation (19, 20), and some studies have reported that IFN-γ is detrimental in asthma pathogenesis (21, 22). We investigated the individual roles of these cytokines using *ifng^–/–^* mice and IL-17A^Cre^ homozygous mice that are deficient in IL-17A (hereafter referred to as *il17a^–/–^* mice). Lack of IFN-γ did not alter neutrophil numbers in BALF, whereas IL-17A deficiency partially suppressed neutrophilia (Figure 2A) without affecting IFN-γ levels (Figure 2B). IL-17A production is regulated by the transcription factor RORγt in various cell types (23, 24). Consistent with this, *rorc^–/–^* mice did not produce IL-17A when exposed to PM_2.5_ (Figure 2C). These mice also developed lower neutrophilia upon exposure to PM_2.5_ (Figure 2D), further confirming the role of IL-17A in neutrophil inflammation.

Next, we sought to identify the cellular source of IL-17A. Flow cytometry analysis revealed that IL-17A was produced by various lymphocytes, including innate lymphoid cells and Th cells, as well as neutrophils in mice exposed to PM_2.5_ (Figure 2E). γδ T cells were the major producers under these conditions, accounting for more than 40% of the total IL-17A production. We also observed a marked increase in the frequencies of γδ T cells and their intrinsic IL-17A–producing capacity in mice exposed to PM_2.5_, compared with controls (Figure 2F). Accordingly, total numbers of γδ T cells and IL-17A–producing γδ T cells were higher in PM_2.5_-exposed mice than unexposed mice (Figure 2G). Although γδ T cells can be induced to produce IFN-γ (25), we did not observe any production of IFN-γ by these cells in the PM_2.5_ model (Supplemental Figure 2A).

We next assessed the impact of γδ T cell depletion using *Tcrd^–/–^* mice. Mice lacking γδ T cells had lower levels of IL-17A and impaired AHR relative to WT mice upon exposure to PM_2.5_ (Figure 2, H and I). Notably, neutrophilia was less severe in *Tcrd^–/–^* mice than in WT counterparts (Figure 2J). Taken together, these data indicate that γδ T cells contribute to the pathogenesis of PM_2.5_ through IL-17A.

### PM_2.5_ activates iNKT cells through induction of apoptotic epithelial cells.

**Figure 3:**
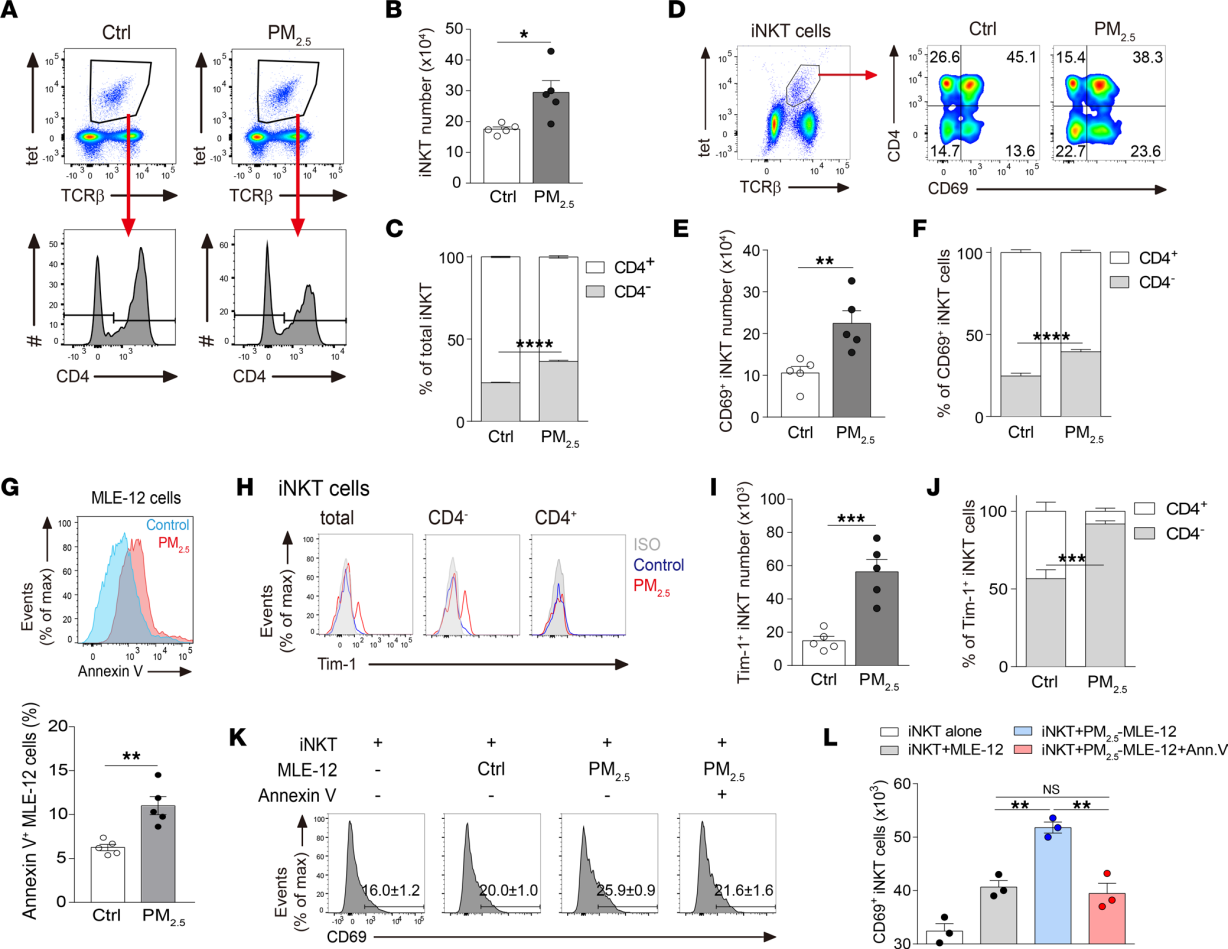
iNKT cell deficiency exacerbates PM_2.5_-induced pulmonary inflammation and IL-17A production. (**A**–**F**) BALB/c (WT) mice received daily i.n. exposure of PM_2.5_ for 3 days and were sacrificed 1 day after the last exposure. (**A**) Representative flow cytometry plot showing CD4^+^ and CD4^–^ iNKT cell subsets, gated from TCRβ^+^CD1d-tetramer^+^ (tet^+^) cells. (**B**) Total number of lung iNKT cells, assessed as in **A**. (**C**) Relative percentages of CD4^+^ and CD4^–^ iNKT cell subsets, assessed as in **A**. (**D**) Representative flow cytometry plot showing CD69 expression on CD4^+^ and CD4^–^ iNKT cell subsets, gated from TCRβ^+^CD1d-tet^+^ cells. (**E**) Total number of CD69^+^ iNKT cells, assessed as in **D**. (**F**) Relative percentages of CD69-expressing CD4^+^ and CD4^–^ iNKT cell subsets, assessed as in **D**. (**G**) Representative histogram (top panel) and frequency (bottom panel) of annexin V^+^ MLE-12 cells after treatment with 300 μg/mL PM_2.5_ for 6 hours. (**H**–**J**) WT mice received daily i.n. exposure of PM_2.5_, as in **A**. (**H**) Representative histogram showing Tim-1 expression on total, CD4^+^, and CD4^–^ iNKT cells. (**I**) Total number of Tim-1^+^ iNKT cells, assessed as in **H**. (**J**) Relative percentages of Tim-1–expressing CD4^+^ and CD4^–^ iNKT cell subsets, assessed as in **H**. (**K**) Flow histogram showing CD69 expression on iNKT cells cocultured with unexposed or PM_2.5_-exposed MLE-12 cells in the presence or absence of annexin V. (**L**) Total CD69^+^ iNKT cells, assessed as in **K**. Data are shown as mean ± SEM from 2 independent experiments (*n* = 5 per group) (**B**–**F**, **I**, and **J**) or mean ± SD from 1 representative experiment (*n* = 3 wells) with consistent findings (**G** and **L**). Statistical analysis was performed using an unpaired 2-tailed *t* test. *P < 0.05, **P < 0.01, ***P < 0.001, and ****P < 0.0001. Ctrl, control; ISO, isotype; Max, maximum.

In addition to increases in γδ T cell numbers upon treatment with PM_2.5_, we also observed an increase in the numbers of lung iNKT cells, particularly the CD4^–^ subset in PM_2.5_-exposed mice (Figure 3, A–C). There was also greater expression of the activation marker CD69 in the overall iNKT cell population in mice exposed to PM_2.5_, compared with controls, and the increase was most significant in the CD4^–^ subset in terms of frequency (Figure 3, D–F). Although iNKT cells can be stimulated to produce IL-17A and IFN-γ (26, 27), neither cytokine was induced by PM_2.5_ (Supplemental Figure 2, B and C).

PM_2.5_ exposure increased pulmonary cell apoptosis in mice (Figure 1E), and PM_2.5_ directly induced apoptosis of epithelial MLE-12 cells in culture (Figure 3G). Apoptotic cells can activate iNKT cells through binding of PtdSer to Tim-1 expressed on the iNKT cell surface (28). Our results show that PM_2.5_ markedly upregulated Tim-1 on the CD4^–^ iNKT cell subset; the CD4^+^ subset did not show any Tim-1 expression (Figure 3, H–J).

To confirm the role of PtdSer/Tim-1 signaling, we cocultured PM_2.5_-exposed MLE-12 cells and iNKT cells in the absence or presence of annexin V, which binds to PtdSer and blocks its recognition (29). As expected, PM_2.5_-exposed MLE-12 cells increased the percentage and number of CD69^+^ iNKT cells in the coculture. Importantly, addition of annexin V impaired iNKT cell activation (Figure 3, K and L). These results suggest that PM_2.5_-induced iNKT cell activation is mediated by the PtdSer–Tim-1 axis.

### iNKT cells protect against PM_2.5_-induced AHR and airway inflammation through suppression of γδ T cells.

**Figure 4:**
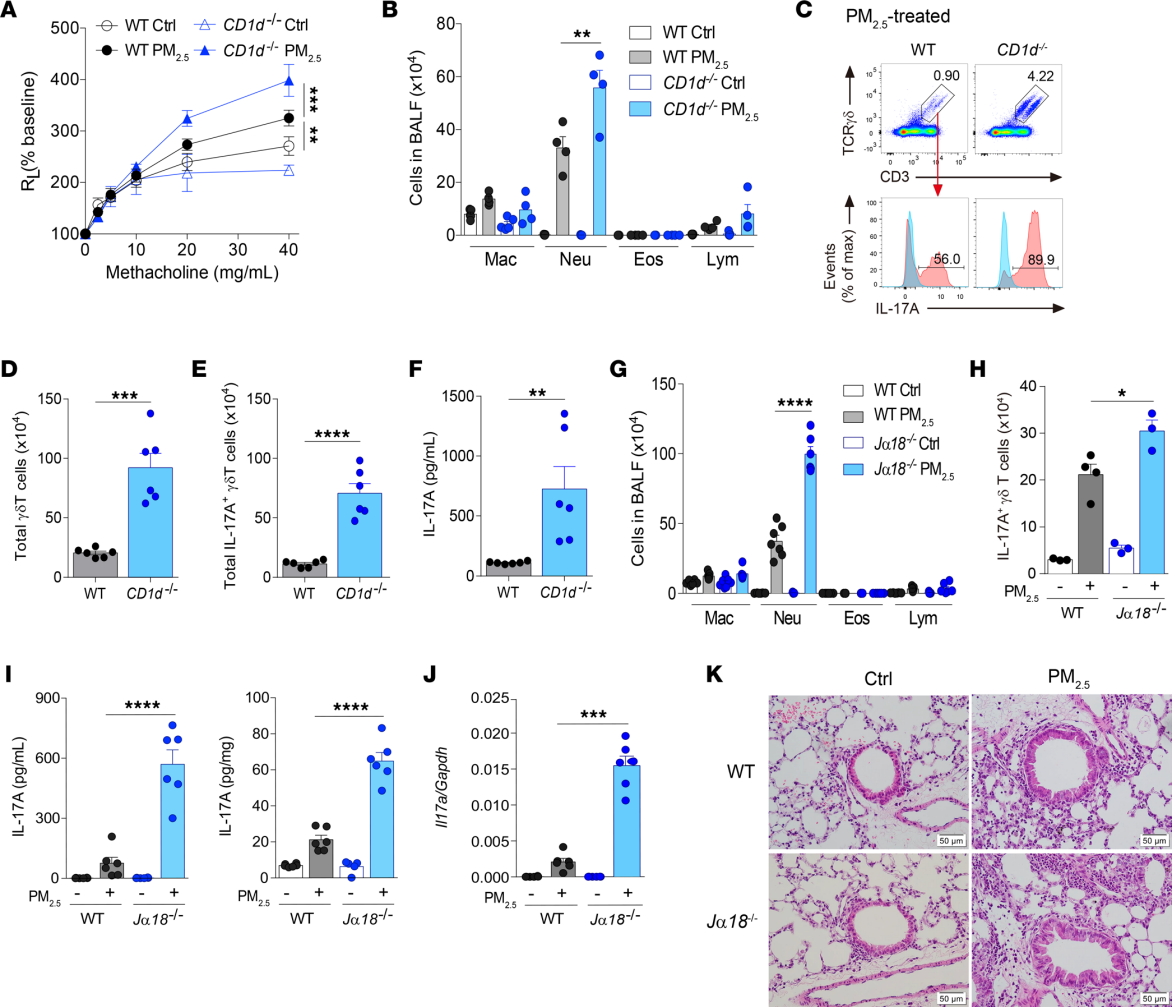
iNKT cell deficiency exacerbates PM_2.5_-induced pulmonary inflammation and IL-17A production. (**A**–**F**) BALB/c (WT) and *CD1d^–/–^* mice received daily i.n. exposure of PM_2.5_ for 3 days and were sacrificed 1 day after the last exposure. (**A**) Lung resistance in response to increasing doses of methacholine. (**B**) Cellular composition in BALF. (**C**) Representative flow cytometry plot showing IL-17A–producing γδ T cells (CD3^+^TCRγδ^+^ cells). Blue solid line: isotype-matched control; red solid line: Ab staining. (**D**) Total lung γδ T cells, assessed as in **C**. (**E**) Total IL-17A–producing γδ T cells, assessed as in **C**. (**F**) IL-17A level in the BALF. (**G**–**K**) WT and *J*α*18^–/–^* mice received daily i.n. exposure of PM_2.5_ for 3 days and were sacrificed 1 day after the last exposure. (**G**) Cellular composition in BALF. (**H**) Total IL-17A^+^ γδ T cells, assessed as in **C**. (**I**) IL-17A level in the BALF (left) and lungs (right). (**J**) *Il17a* levels in the lungs. (**K**) Representative images of H&E-stained histologic sections of lung tissues and quantification of bronchial thickness. (Scale bars: 50 μm; original magnification, ×40.) Data are shown as mean ± SEM from 2 independent experiments (*n* = 3–7 per group). Statistical analysis was performed using 2-way ANOVA (**A**), 1-way ANOVA (**B** and **G**–**J**), or an unpaired 2-tailed *t* test (**D**–**F**). *P < 0.05, **P < 0.01, ***P < 0.001, and ****P < 0.0001. Ctrl, control; Eos, eosinophil; Lym, lymphocyte; Mac, macrophage; Max, maximum; Neu, neutrophil; R_L_, lung resistance.

The role of iNKT cells in asthma is controversial, with some studies showing protective function and others reporting detrimental roles of these innate-like lymphocytes (30, 31). To determine the role of iNKT cells in PM_2.5_-induced AHR and neutrophilic inflammation, we used *CD1d^–/–^* mice that lack all NKT cells (both type I and II). Interestingly, NKT cell–deficiency exacerbated AHR and neutrophilia detected in BALF (Figure 4, A and B). Flow cytometry analysis revealed higher frequencies of γδ T cells with enhanced intrinsic potential to produce IL-17A in PM_2.5_-exposed *CD1d^–/–^* mice compared with WT mice (Figure 4C). In support of this finding, total numbers of lung γδ T cells and IL-17A^+^ γδ T cells were higher in PM_2.5_-exposed mice lacking iNKT cells than in PM_2.5_-exposed WT mice (Figure 4, D and E). Of note, naive γδ T cells from iNKT cell–deficient mice also had enhanced capacity to produce IL-17A, compared with their WT counterparts (Supplemental Figure 3). We also observed substantially higher levels of IL-17A in BALF in PM_2.5_-exposed *CD1d^–/–^* mice than in PM_2.5_-exposed WT mice (Figure 4F).

To confirm the role of iNKT cells, we examined the effects of PM_2.5_ on *J*α*18^–/–^* mice, which lack only iNKT cells. The findings in PM_2.5_-treated *J*α*18^–/–^* mice recapitulated those in *CD1d^–/–^* mice in terms of augmented neutrophilic inflammation and increased IL-17A^+^ γδ T cells (Figure 4, G and H). Similarly, IL-17A levels were higher in *Jα18^–/–^* mice than in WT mice after exposure to PM_2.5_ at both protein and mRNA levels (Figure 4, I and J). Furthermore, bronchial epithelial hypertrophy and cellular infiltration were more severe in mice lacking iNKT cells (Figure 4K). Notably, Th1 and Th2 responses were similar upon PM_2.5_ treatment in *Jα18^–/–^* and WT mice (Supplemental Figure 4). The stronger Th17 response and more intense pulmonary pathology in iNKT cell–deficient mice upon PM_2.5_ exposure suggest that iNKT cells moderate PM_2.5_-induced pathology.

### Reconstitution of CD4^–^ iNKT cell subset alleviates PM_2.5_-induced airway inflammation.

**Figure 5:**
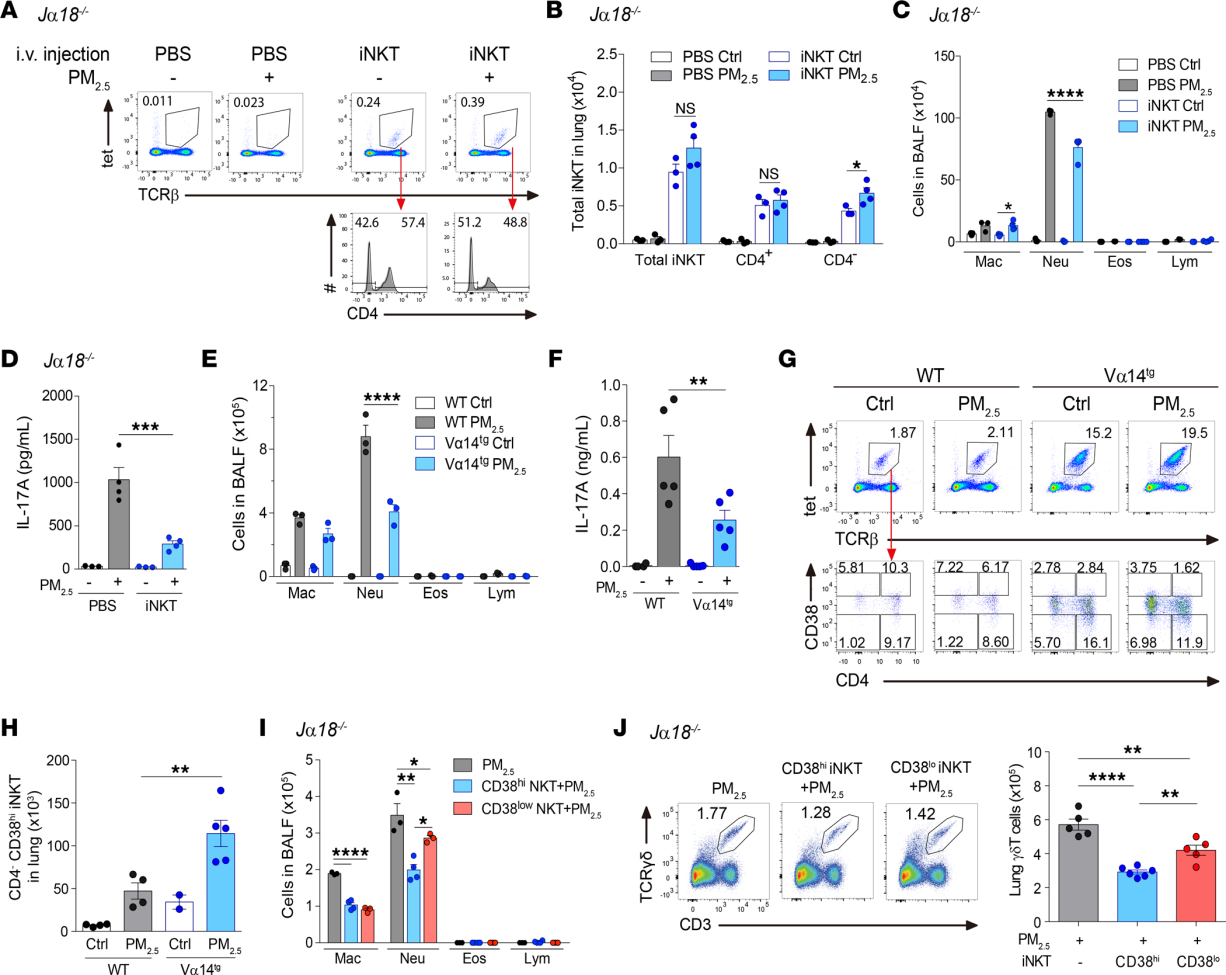
Reconstitution of CD4^–^ iNKT cells attenuates PM_2.5_-induced pulmonary inflammation and IL-17A production. (**A**–**D**) Splenic iNKT cells were sorted from naive Vα14^Tg^ mice and i.v. injected into *J*α*18^–/–^* mice 30 minutes before the first exposure of PM_2.5_. In mock groups, mice received PBS instead. Mice received daily i.n. exposure of PM_2.5_ for 3 days and were sacrificed 1 day after the last exposure. (**A**) Validation of iNKT cell reconstitution by flow cytometry. (**B**) Absolute numbers of total, CD4^–^, and CD4^+^ iNKT cell subsets, assessed as in **A**. (**C**) Cellular composition in BALF. (**D**) IL-17A level in BALF. (**E**–**H**) BALB/c (WT) and Vα14^Tg^ mice received daily i.n. exposure of PM_2.5_, as in **A**. (**E**) Cellular composition in BALF. (**F**) IL-17A level in BALF. (**G**) Representative flow cytometry plot showing CD4^–^CD38^hi^, CD4^+^CD38^hi^, CD4^–^CD38^lo^, and CD4^+^CD38^lo^ iNKT cell subsets. (**H**) Total number of CD4^–^CD38^hi^ iNKT cell subset, assessed as in **G**. (**I** and **J**) CD4^+^CD38^hi^ and CD4^+^CD38^lo^ iNKT cells were sorted from naive Vα14^Tg^ mice and i.v. injected into *J*α*18^–/–^* mice 30 minutes before the first exposure of PM_2.5_. Mice received daily i.n. exposure of PM_2.5_, as in **A**. (**I**) Cellular composition in BALF. (**J**) Representative flow cytometry plot showing IL-17A–producing γδ T cells (CD3^+^TCRγδ^+^ cells) (left) and total lung γδ T cells (right). Data are shown as mean ± SEM from 2 independent experiments (*n* = 2–6 per group). Statistical analysis was performed using 1-way ANOVA. *P < 0.05, **P < 0.01, ***P < 0.001, and ****P < 0.0001. Ctrl, control; Eos, eosinophil; Lym, lymphocyte; Mac, macrophage; Neu, neutrophil.

To further confirm the protective role of iNKT cells, we performed adoptive transfer of splenic iNKT cells into *J*α*18*^–*/*–^ mice. Efficiency of lung iNKT cell reconstitution was validated by flow cytometry. Although the population size of reconstituted iNKT cells in the lung was equivalent to only a quarter of the endogenous pulmonary iNKT cells in WT mice (~0.25% and ~1% of the total CD45^+^ population, respectively), these cells had a response to PM_2.5_ that was similar to the endogenous iNKT cells in WT mice with PM_2.5_ exposure resulting in accumulation of CD4^–^ iNKT cells in the lungs (Figure 5, A and B). Importantly, reconstitution of iNKT cells attenuated neutrophilia and IL-17A production (Figure 5, C and D). Likewise, Vα14^Tg^ mice, a mouse strain with greatly increased iNKT cell numbers compared with WT mice (32), developed less severe neutrophilia and produced less IL-17A than did WT mice (Figure 5, E and F). These data reinforce the notion that iNKT cells protect against PM_2.5_-induced lung pathogenesis.

A recent study reported a suppressive CD4^–^ iNKT cell subset with high CD38 expression (31). Our flow cytometry analysis revealed a significant increase in the CD38^hi^CD4^–^ iNKT cell subset upon exposure to PM_2.5_ (Figure 5, G and H). Therefore, we hypothesized that the CD38^hi^CD4^–^ iNKT cell subset is responsible for protection against the effects of PM_2.5_. To test this, we sorted CD38^hi^CD4^–^ and CD38^lo^CD4^–^ iNKT cell subsets and adoptively transferred these cells into *J*α*18*^–*/*–^ mice. In contrast to our hypothesis, we found that both subsets suppressed PM_2.5_-induced neutrophilia and reduced γδ T cell frequency and total numbers, although the CD38^hi^CD4^–^ iNKT cell subset showed slightly stronger suppression (Figure 5, I and J). Taken together, these data imply that both CD38^hi^CD4^–^ and CD38^lo^CD4^–^ iNKT cell subsets suppress airway neutrophilia and γδ T cell accumulation in the lungs of PM_2.5_-treated mice.

### iNKT cells directly suppress γδ T cell function through PD-1/PD-L1 interaction.

**Figure 6:**
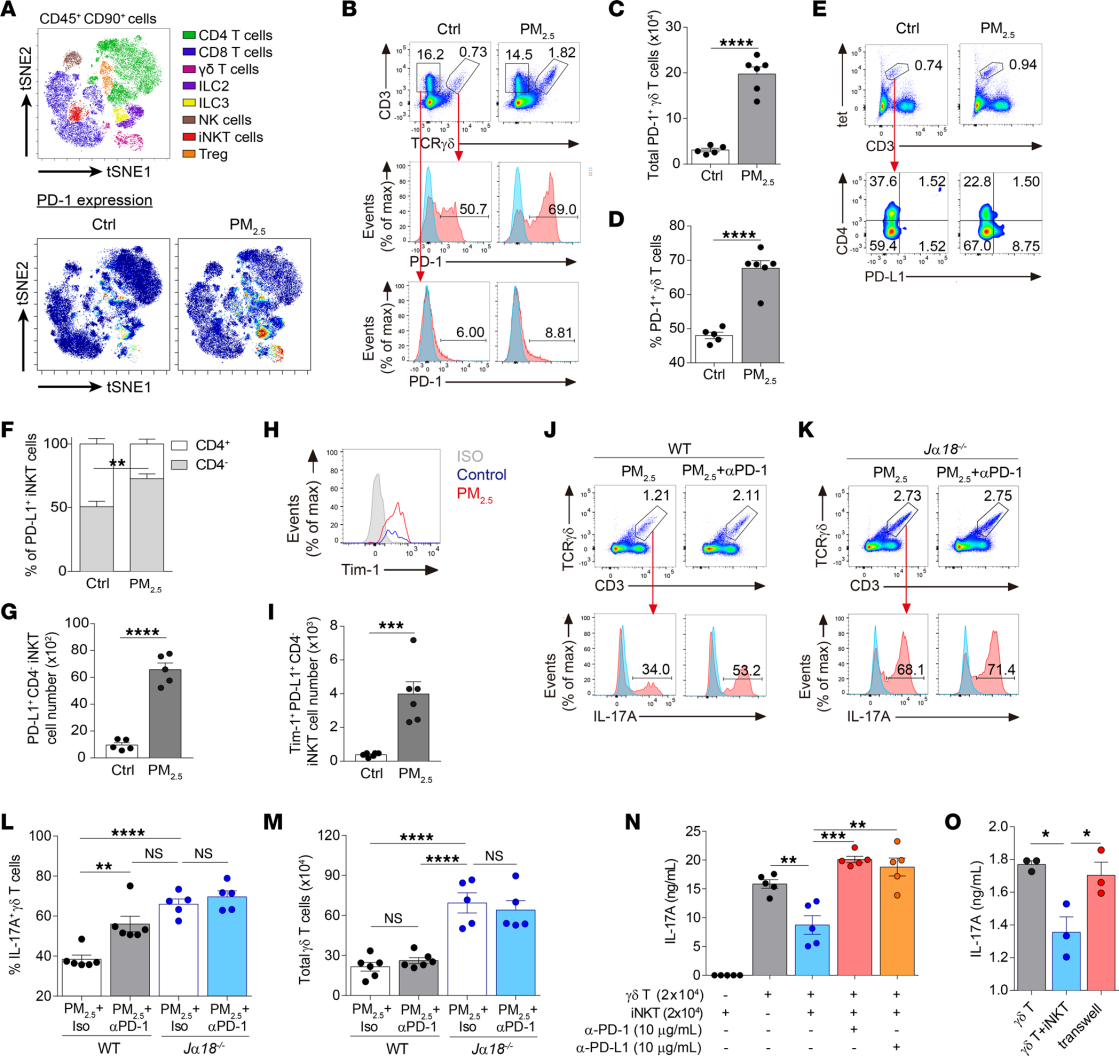
iNKT cells suppress IL-17A production by γδ T cells through PD-1/PD-L1 interaction. (**A**–**I**) C57BL/6 (WT) mice received daily i.n. exposure of PM_2.5_ for 3 days and were sacrificed 1 day after the last exposure. (**A**) A viSNE map showing PD-1 expression in various lymphocytes. (**B**) Representative flow cytometry plot showing PD-1 expression on γδ T cells (CD3^+^TCRγδ^+^ cells) and CD3^+^TCRγδ^–^ T cells. (**C** and **D**) Total number (**C**) and frequency (**D**) of PD-1^+^ γδ T cells, assessed as in **B**. (**E**–**G**) Representative flow cytometry plot (**E**), relative percentages (**F**), and total number (**G**) of PD-L1–expressing CD4^+^ and/or CD4^–^ iNKT cells. (**H** and **I**) Representative histogram (**H**) and total number (**I**) of Tim-1^+^PD-L1^+^CD4^–^ iNKT cells, assessed as in **E**. (**J**–**M**) WT and *J*α*18*^–/–^ mice were exposed to PM_2.5_ as in **A**. Anti–PD-1 or isotype control were administered 1 day before and 1 day after the first exposure to PM_2.5_. (**J** and **K**) Representative flow cytometry plot showing IL-17A^+^ γδ T cells in WT (**J**) and *J*α*18^–/–^* mice (**K**). (**L** and **M**) Frequency of IL-17A–producing (**L**) and total (**M**) lung γδ T cells, assessed as in **J** and **K**. (**N** and **O**) Levels of IL-17A in culture supernatant of γδ T cells cocultured with CD4^–^ iNKT cells under IL-23 (50 ng/mL) and IL-1β (50 ng/mL) stimulation for 48 hours. (**N**) Anti–PD-1 (10 μg/mL) or anti–PD-L1 (10 μg/mL) was added to the coculture system. (**O**) Coculture was performed in a Transwell system. Data are expressed as mean ± SEM from 2 independent experiment (*n* = 5–6 per group) (**A**–**M**) or mean ± SD from 1 representative experiment (*n* = 5 wells for **N**; *n* = 3 wells for **O**) with consistent findings (**N** and **O**). In the histograms, the blue solid line indicates the isotype-matched control, and the red solid line indicates Ab staining. Statistical analysis was performed using an unpaired 2-tailed *t* test (**C**–**I**) or 1-way ANOVA (**L**–**O**). *P < 0.05, **P < 0.01, ***P < 0.001, and ****P < 0.0001. Ctrl, control; ISO, isotype; Max, maximum.

Upon activation, γδ T cells transiently upregulate various inhibitory receptors, such as PD-1 and cytotoxic T lymphocyte associated protein 4 (33). Mass cytometry by TOF (CyTOF) analysis revealed substantial increases in PD-1 expression in group 2 innate lymphoid cells, group 3 innate lymphoid cells, and γδ T cells after exposure to PM_2.5_ (Figure 6A). Likewise, flow cytometry analysis showed that there were significant increases in both frequencies and total numbers of PD-1^+^ γδ T cells in PM_2.5_-exposed mice compared with control mice (Figure 6, B–D). Notably, other T cell types (e.g., CD3^+^TCRγδ^–^ cells) did not show any significant increases in PD-1 expression after exposure to PM_2.5_ (Figure 6B).

Both PD-1 and PD-L1 are expressed on splenic iNKT cells (34, 35). Consistent with this, a small fraction of CD4^+^ and CD4^–^ iNKT cell subsets expressed PD-1 at steady state; however, PD-1 expression decreased after PM_2.5_ exposure (Supplemental Figure 5, A–C). In contrast, exposure to PM_2.5_ markedly increased PD-L1 expression on iNKT cells, particularly the CD4^–^ subset (Figure 6, E and F). Notably, the frequencies of PD-L1–expressing CD38^hi^ and CD38^lo^ subsets did not show significant differences (Supplemental Figure 5, D and E), suggesting that the inherent suppressive capacity of the CD38^hi^ subset likely mediates the stronger inhibitory effect of this subset, as seen in Figure 5, I and J. Consistent with the frequency, numbers of PD-L1^+^CD4^–^ iNKT cells were also increased (Figure 6G). To examine whether the observed increment was due to local cell proliferation or recruitment of circulating CD4^–^ iNKT cells, we injected CFSE i.v. into PM_2.5_-exposed mice, which labeled up to 85% of iNKT cells in the circulation (Supplemental Figure 6A). Cell proliferation was determined by Ki-67 labeling. Circulating CD4^–^ iNKT cells (CFSE^+^ cells) constituted approximately 30% of total PD-L1^+^CD4^–^ iNKT cells but were nonproliferative (Ki-67^–^). Proliferating tissue-resident iNKT cells (CFSE^–^Ki-67^+^ cells), on the other hand, accounted for approximately 10% of the total PD-L1–expressing CD4^–^ iNKT cells (Supplemental Figure 6B). These data indicate that the increase in PD-L1^+^CD4^–^ iNKT cell numbers is attributed to both recruitment of circulating CD4^–^ iNKT cells and, to a lesser extent, proliferation of tissue-resident CD4^–^ iNKT cells.

In addition to the aforementioned findings, we also noted that the PD-L1–expressing subset coexpressed Tim-1 (Figure 6H), and numbers of these cells were substantially elevated in PM_2.5_-exposed mice (Figure 6I). On these bases, we postulated that PD-1/PD-L1 signaling is involved in iNKT cell–mediated suppression of γδ T cell function. To determine whether iNKT cells modulate γδ T cell function through the PD-1/PD-L1 interaction, we administered anti–PD-l Ab to mice to block PD-1/PD-L1 signaling. Treatment with anti–PD-1 Ab boosted the intrinsic production of IL-17A by γδ T cells but did not increase the total number of these cells; this effect was not seen in *J*α*18*^–*/*–^ mice (Figure 6, J–M). Together, these results indicate that iNKT cells are required for PD-1–mediated inhibition of γδ T cell function.

To examine whether this inhibition is direct, we cocultured CD4^–^ iNKT cells and γδ T cells in the presence or absence of anti–PD-1 or anti–PD-L1 neutralizing Abs. Consistent with the suppressive role of iNKT cells, addition of the CD4^–^ iNKT cell subset reduced IL-17A levels in the culture supernatant, and treatment with either anti–PD-1 or anti–PD-L1 reversed this inhibition (Figure 6N). Moreover, iNKT cell–mediated suppression was abolished in a Transwell culture system (Figure 6O), indicating a requirement for cell-to-cell contact. Taken together, our results show that PD-1/PD-L1 signaling plays a crucial role in iNKT cell–mediated suppression of IL-17A production by γδ T cells.

## Discussion

Exposure to PM_2.5_ is associated with increased frequency of exacerbations of and hospitalizations for asthma. Although it is known that PM_2.5_ stimulates a wide range of immune effector responses, the exact immunological factors that contribute to pulmonary inflammation and development of asthma remain incompletely understood. Here, we showed that PM_2.5_ elicits AHR and airway inflammation characterized by acute neutrophilic inflammation, epithelial cell hypertrophy, and mixed Th1/Th17 responses. We demonstrated that these pathological features are mediated by IL-17A, which is produced mainly by γδ T cells. Concomitantly, PM_2.5_-induced epithelial cell apoptosis exposes PtdSer on apoptotic cell surfaces and activates the CD4^–^ iNKT cell subset through interaction with Tim-1 expressed on iNKT cells. Importantly, we showed that this CD4^–^ iNKT cell subset upregulates PD-L1 expression upon activation and plays a protective role by counter-regulating IL-17A production by γδ T cells through PD-1/PD-L1 signaling.

PM_2.5_ aggravates asthma-like symptoms in various allergic asthma models induced by cockroach extract and house dust mites through Th17 cell–driven inflammation (36, 37). The effects of PM_2.5_ alone on AHR development and lung inflammation had not been thoroughly studied, however. Our results indicated that PM_2.5_ alone rapidly induced both Th1 and Th17 cytokines with concomitant increases in neutrophilic inflammation and lung resistance as early as day 1 after exposure. As also shown in previous studies (36, 37), we found that IL-17A is the effector cytokine that drove lung pathology and AHR upon exposure to PM_2.5_. However, we found that γδ T cells are the dominant early source of this cytokine rather than Th17 cells, indicating that γδ T cells are the first responders during short-term PM_2.5_ exposure. This is consistent with their role as initiators of immune responses, which result from their rapid, innate-like properties (38). We also detected substantial increases in the mRNA and protein levels of IL-1β and IL-23, which have been shown to stimulate IL-17A production by γδ T cells (15). Because γδ T activation does not require antigen presentation by MHC molecules (39), it is likely that these cells are activated by these proinflammatory cytokines.

T cell coinhibitory molecules such as PD-1 help keep T cell responses in check to avoid self-damage (40). In this study, we found that CD4^–^ iNKT cells suppressed IL-17A production by γδ T cells through PD-1/PD-L1 signaling. To our knowledge, this is the first study to report suppression of γδ T cell function by iNKT cells through coinhibitory molecule signaling. Although it was reported that iNKT cells can suppress IL-17A production by γδ T cells in *Salmonella enterocolitis*–induced reactive arthritis (41), the mechanism involved was not elucidated. Moreover, several studies have also shown that PD-1 signaling attenuates IL-17A production by γδ T cells under various disease conditions (42, 43); however, the cellular source of the PD-1 ligand was not defined. iNKT cells express PD-L1 but not PD-L2 ligand (34). We found that PM_2.5_ upregulated PD-L1 expression on the CD4^–^ iNKT cell subset and caused increased PD-1 expression on γδ T cells and that PD-1/PD-L1 signaling regulated the intrinsic IL-17A–producing ability of γδ T cells. Nonetheless, anti–PD-1 did not completely restore the IL-17A production potential of γδ T cells to the levels seen in *J*α*18*^–*/*–^ mice, indicating the involvement of other mechanisms that remains to be defined. PD-L1 is expressed on various immune cells, including macrophages, DCs, and activated B and T cells (44). Moreover, PD-1 can also interact with PD-L2, which is expressed primarily on macrophages and DCs (45). Because blockade of PD-1 signaling in mice lacking iNKT cells did not further enhance intrinsic IL-17A production by γδ T cells, the expression of PD-1 ligands on other cells does not affect γδ T cell function.

Our analyses also revealed that CD4^–^ iNKT cells suppressed γδ T cell expansion upon exposure to PM_2.5_. This effect was independent of PD-1 signaling, given that anti–PD-1 treatment did not enhance the frequency or total number of lung γδ T cells. A CD38^hi^CD4^–^ iNKT cell subset was recently shown to suppress CD4^+^ T cell proliferation and may serve as a marker to distinguish suppressive iNKT cells (31). Although PM_2.5_ induces this particular subset, the suppressive effect of CD4^–^ iNKT cells is not limited to the CD38^hi^ population, because CD38^lo^ iNKT cells also suppress lung γδ T cell expansion, although to a lesser degree. Exactly how PM_2.5_-activated CD4^–^ iNKT cells inhibit γδ T cell expansion remains to be determined. Of note, we also observed a marked increase in IFN-γ production in mice exposed to PM_2.5_; IFN-γ suppresses Th17 differentiation and IL-17A production in experimental models of arthritis and autoimmune encephalomyelitis (46, 47). Moreover, IFN-γ–producing iNKT cells are associated with protection from airway inflammation (48). We did not detect IFN-γ production by iNKT cells in response to PM_2.5_, and, importantly, IFN-γ deficiency did not alter the pathological outcome of PM_2.5_, indicating that this cytokine is not necessary for the function of iNKT cells in the response to PM_2.5_.

We found that PM_2.5_ upregulated Tim-1 expression on the suppressive PD-L1^+^CD4^–^ iNKT cell subset. Tim-1 is a member of the family of T cell immunoglobulin and mucin domain family of proteins and is expressed on mast cells, macrophages, activated Th2 cells, and iNKT cells (49–52). Previous studies have shown that Tim-1 mediates iNKT cell activation through recognition of PtdSer on apoptotic cells (28, 53). Consistent with these findings, we found that PM_2.5_ induced bronchial epithelial cell apoptosis and that blockade of PtdSer exposed on PM_2.5_-treated epithelial cells with annexin V prevents iNKT cell activation. Despite functioning as a T cell costimulatory molecule, Tim-1 signaling alone can stimulate iNKT cell proliferation and cytokine production (52). It is unclear whether this was the case in our study or whether PM_2.5_ also triggers CD1d-mediated activation, because the lipid antigens produced upon PM_2.5_ exposure have yet to be identified. A recent study showed that ozone exposure can lead to iNKT cell activation, likely through recognition of oxidized lipids (54). Chronic exposure to PM_2.5_ can also induce formation of oxidized lipids (55), but whether the same mechanism occurs in our acute model remains to be determined.

In conclusion, we demonstrate that PM_2.5_ exposure activated a protective subset of iNKT cells that suppressed airway inflammation and AHR by inhibiting γδ T cell expansion and function. Mechanistically, PM_2.5_ triggers epithelial cell apoptosis, which, in turn, activates the CD4^–^ iNKT cell subset through PtdSer-mediated Tim-1 signaling. Activated Tim-1^+^CD4^–^ iNKT cells upregulated PD-L1 expression on their surface and suppressed IL-17A production by γδ T cell through PD-1/PD-L1 interaction. Therefore, CD4^–^ iNKT cells could serve as a potential target for immunotherapy, and strategies to exploit their function, such as the use of Tim-1–activating monoclonal Abs, should be explored as a possible therapeutic option for management of nonallergic asthma.

## Methods

### Mice.

BALB/c and C57BL/6 mice were purchased from Taiwan National Laboratory Animal Center. Vα14Tg mice were purchased from Jackson Laboratory. IL-17A^Cre^ and Rorc^eGFP^ mice were provided by Jr-Wen Shui (Academia Sinica). Homozygous IL-17A^Cre^ and Rorc^eGFP^ mice were used as IL-17A– and RORγt-deficient mice, respectively. *J*α*18^–/–^* and *CD1d^–/–^* mice were provided by Masaru Taniguchi (RIKEN Center for Integrative Medical Sciences, Yokohama, Japan); *Tcrd^–/–^* mice were obtained from Leo Yung-Ling Lee (Academia Sinica); *Il13^–/–^* mice were obtained from Andrew J. McKenzie (Medical Research Council Laboratory of Molecular Biology, Cambridge, United Kingdom); and *Ifng^–/–^* mice were provided by Nan-Shih Liao (Academia Sinica). Experiments were performed with age- and sex-matched mice.

### In vivo administration of PM_2.5_ or neutralizing Ab.

Mice received 200 μg of PM_2.5_ (SRM2786, Sigma-Aldrich) i.n. once a day for 3 days. Mice were sacrificed 1 day after the last exposure unless specified otherwise. To block the PD-1/PD-L1 interaction, anti–PD-1 (RMP1-14) was administered at 100 μg/mouse 1 day before and 1 day after the first exposure to PM_2.5_. Rat IgG2b.κ (100 μg/mouse) was given as the isotype control. Both Abs were purchased from Bio X Cell.

### In vivo CFSE labeling.

To track migration of iNKT cells into the lung, blood cells were labeled with CFSE (Cayman Chemical) through i.v. injection, as previously described (56). Briefly, mice were exposed to PM_2.5_ daily through the i.n. route for 3 days. Mice were injected with CFSE 2 μg/g mouse weight after the second exposure to PM_2.5_ and sacrificed 1 day after the last exposure to PM_2.5_.

### Measurement of airway AHR.

Mice were anesthetized with pentobarbital (Sigma-Aldrich) at 100 mg/kg body weight. AHR was determined by measuring airway resistance in response to increasing doses of methacholine (Sigma-Aldrich), using the FinePointe RC system (Buxco Research Systems).

### BALF collection for differential cell counting and ELISA.

Mouse trachea was exposed and lungs were lavaged twice with 2% FCS in PBS using a 20-gauge i.v. catheter (Terumo). Cells were obtained from BALF by centrifugation at 400*g* for 5 minutes at 4°C. RBCs were lysed with RBC lysis buffer (Omics Bio), and cells were spun onto slides and stained with Diff-Quick solution (Polysciences, Inc.). The BALF cellular profile was assessed by differential cell counting. For ELISA measurement, lavage was performed with PBS supplemented with protease inhibitor III (Merck), phosphatase inhibitor II (Merck), and phosphatase inhibitor III (Merck).

### Isolation of mononuclear cells from the blood.

Blood was drawn from the heart using a 27-gauge needle and immediately mixed with PBS solution containing EDTA (final concentration, 4 mM). The mixed blood solution was overlaid on an equal volume of Histopaque-1077 (Sigma-Aldrich) and centrifuged at 400*g* for 30 minutes without a brake at room temperature. The opaque interface containing mononuclear cells was collected and washed twice with 2% FCS and PBS.

### Lung processing for flow cytometry and CyTOF.

Whole lungs were minced and digested in DMEM containing 0.1% (vol/vol) DNase I (Worthington Biochemicals) and 1.6 mg/mL collagenase IV (Worthington Biochemicals) at 37°C. After 30 minutes of digestion, tissue aggregates were dissociated with an 18-gauge needle and lung tissues were further incubated at 37°C for 15 minutes. Tissues were filtered through a 70 μm mesh to obtain single-cell suspensions. RBCs were removed from the cell suspensions using ACK lysing buffer (Gibco Laboratories).

### Flow cytometry.

Single-cell suspensions were stained with fixable viability dye for 30 minutes at 4°C, followed by preincubation with anti–mouse CD16/32 blocking Ab (1:100 dilution, TruStain fcX, BioLegend) for 10 minutes at 4°C. After Fc blocking, cells were stained with surface antigens at 4°C for 30 minutes. For intracellular staining of transcription factors and cytokines, cells were fixed and permeabilized using the Foxp3 transcription factor staining kit (Thermo Fisher Scientific). For intracellular cytokine staining, cells were prestimulated with 100 ng/mL phorbol 12-myristate 13-acetate (Sigma-Aldrich) and 1 μg/mL ionomycin (Sigma-Aldrich) for 4 hours in the presence of GolgiStop (BD Biosciences) during the last hour of incubation. Data acquisition was performed on an LSR II (BD Biosciences) and analyzed with FlowJo v10 (Tree Star, Inc.). For flow cytometry analysis, TCRγδ (GL3), IL-17A (17F3), TCRβ (H57-597), CD3 (145-2C11), CD45 (30-F11), CD11b (M1/70), CD11c (N418), FcεRI (MAR-1), CD49b (DX5), CD19 (6D5), F4/80 (BM8), Ly6G (1A8), CD4 (GK1.5), and GATA3 (16E10A23) were purchased from BioLegend. Fixable viability dye eFluor 780, Thy1.2 (53-2.1), Ki-67 (SolA15), and RORγt (AFKJS-9) were purchased from Thermo Fisher Scientific. CD1d-tetramer (PE or BV421 labeled) was obtained from the NIH tetramer core facility (Emory University).

### Sample processing for CyTOF analysis.

After lung digestion, single-cell suspensions were washed once with cell-staining medium (CSM; PBS with 0.5% BSA and 0.02% sodium azide). Fc receptors were blocked with anti–mouse CD16/32, and cells were stained with a surface Ab cocktail for 1 hour. Cells were then washed with CSM and stained with cisplatin (Sigma-Aldrich) at a final concentration of 25 μM for 1 minute at room temperature to label dead cells. After quenching by adding an equal volume of complete medium, cells were fixed and permeabilized using the Foxp3 transcription factor staining kit (Thermo Fisher Scientific) and then stained with an intracellular Ab cocktail. Cells were washed twice with CSM and stained for DNA with Cell-ID Intercalator-Ir (^191^Ir and ^193^Ir; Fluidigm). Samples were resuspended in MilliQ water containing EQ Four Element Calibration Beads (Fluidigm) for normalization.

### CyTOF analysis.

Sample acquisition was performed on a CyTOF2 instrument (Fluidigm). Raw flow cytometry standardfiles acquired from CyTOF2 machine were normalized using the Fluidigm Helios software (Fluidigm). Normalized data were analyzed and visualized using viSNE and FlowSOM (Cytobank). The Abs and gating strategies used for CyTOF analysis are listed in Supplemental Tables 1 and 2, respectively.

### iNKT and γδ T cell sorting.

Total iNKT cells, CD38^hi^CD4^–^ iNKT cells, and CD38^lo^CD4^–^ iNKT cells were sorted from the spleens of Vα14Tg mice. Spleens were harvested, minced, and dispersed into single cells in 2% FCS in PBS. RBCs were lysed and B cells were removed by incubating splenocytes in AffiniPure goat anti–mouse IgG plus IgM (heavy and light chains) for 15 minutes at room temperature. Cells were then stained with surface Abs for 30 minutes at 4°C. Total iNKT cells were sorted as CD45^+^TCRβ^+^CD1d-tetramer^+^ cells, whereas CD38^hi^CD4^–^ and CD38^lo^CD4^–^ iNKT cell subsets were sorted as CD45^+^TCRβ^+^CD1d-tetramer^+^CD38^hi^CD4^–^ cells and CD45^+^TCRβ^+^CD1d-tetramer^+^CD38^lo^CD4^–^ cells, respectively.

To obtain a sufficient number of γδ T cells, mice were pretreated with murine recombinant IL-1β and IL-23 (both at 0.1 μg/mouse) daily for 3 days. Mice were sacrificed 4 days after the last treatment. Lungs were digested as described above, and mononuclear cells were obtained using a 1-step density gradient centrifugation in 33% Percoll (GE Healthcare, now Cytiva). γδ T cells were sorted as CD45^+^CD3^+^TCRγδ^+^ cells. Cell sorting was performed with a FACS Aria cell sorter (BD Biosciences) with a sorting purity of greater than 95%.

### Adoptive transfer.

Total iNKT cells (5 × 10^6^ cells/mouse), CD38^hi^CD4^–^ iNKT cells (2 × 10^5^ cells/mouse), and CD38^lo^CD4^–^ iNKT cells (2 × 10^5^ cells/mouse) were sorted from the spleens of Vα14Tg mice and injected i.v. into *J*α*18^–/–^* mice 30 minutes before the first exposure to PM_2.5_. Mice were given PM_2.5_ i.n. daily for 3 days and were sacrificed 1 day after the last exposure. Reconstitution of cells in the lungs was confirmed by flow cytometry analysis.

### iNKT and γδ T cell coculture.

Sorted lung γδ T cells (2 × 10^4^ cells in 96-well round-bottom plate) were rested overnight in RPMI 1640 supplemented with IL-2 (10 ng/mL), IL-23 (10 ng/mL), IL-1β (10 ng/mL), and IL-7 (20 ng/mL). Culture medium was replaced the next day with RPMI 1640 containing IL-2 (10 ng/mL), IL-7 (20 ng/mL), IL-23 (50 ng/mL), and IL-1β (50 ng/mL), and sorted splenic iNKT cells (2 × 10^4^ cells/well) were added in the absence or presence of anti–PD-1 (RMP1-14), anti–PD-L1 (10F.9G2) Ab, or the relevant isotype control Abs (all at 10 μg/mL). Cells were cocultured for another 48 hours. For the Transwell system, γδ T cells (2 × 10^4^ cells/well) were cultured in a 24-well dish, whereas iNKT cells (2 × 10^4^ cells/well) were cultured in 0.4 μm culture inserts (Grenier Bio-One) in complete RPMI 1640 medium containing IL-2 (10 ng/mL), IL-7 (20 ng/mL), IL-23 (50 ng/mL), and IL-1β (50 ng/mL) for 48 hours.

### MLE-12 and iNKT cell coculture.

The MLE-12 cell line (American Type Culture Collection) was seeded at a density of 2 × 10^4^ cells/well and pretreated with PM_2.5_ (300 μg/mL) for 6 hours. Sorted splenic iNKT cells (1 × 10^5^ cells/well) were cocultured with either unexposed or PM_2.5_-exposed MLE-12 cells in the presence or absence of annexin V (10 μg/mL) for 48 hours.

### Lung histopathology staining.

For tissue block, perfused lungs were fixed with 4% paraformaldehyde (Merck) overnight. Fixed lungs were then dehydrated sequentially with 30%, 50%, and 70% ethanol, followed by paraffin embedding. Paraffin-embedded lung sections were stained with H&E. Images were acquired with an Olympus CX31 microscope (Olympus Corp.).

### TUNEL assay.

TUNEL assays on lung sections were performed following the instructions supplied with the in situ Cell Death Detection Kit (Roche). Lung tissue sections were dewaxed with xylene and rehydrated sequentially with 100%, 90%, 80%, and 70% ethanol. Permeabilization was performed by incubating slides in permeabilization solution (0.1% Triton X-100 in 0.1% sodium citrate) at room temperature for 10 minutes. Slides were rinsed with PBS twice and stained with 50 μL of TUNEL reaction mix in humidified chamber at 37°C for 1 hour. Slides were rinsed with PBS and mounted with UltraCruz Aqueous Mounting Medium with DAPI (Santa Cruz Biotechnology).

### Lung protein extraction for ELISA.

Lung homogenates were extracted in RIPA lysis buffer containing 0.02 M HEPES-HCl at pH 7.4, 0.15 M NaCl, 1 mM CaCl_2_, 1 mM MgCl_2_, 1% NP40, and 0.5% sodium deoxycholate, and protease inhibitor III and phosphatase inhibitors II and III (Merck). Lung homogenates were ultrasonicated in Bioruptor Sonication System (Diagenode, UCD-300) for 10 cycles with 30-second rest and 30-second sonication per cycle. The resulting sonicated homogenates were then centrifuged at 12,000*g* for 10 minutes at 4°Cto remove cell debris. Supernatants were collected for protein detection by ELISA.

### ELISA.

Cytokines were quantified by ELISA using mouse IL-17A, IFN-γ, IL-1β, IL-12B (p40), and IL-23 ELISA MAX Standard kits purchased from BioLegend and a mouse IL-18 kit purchased from eBioscience.

### Quantitative real-time PCR.

Total lung RNA was converted to cDNA using the high-capacity cDNA reverse transcription kit (Applied Biosystems). Quantitative real-time PCR (qPCR) was performed with Labstar SYBR qPCR kit (TAIGEN Bioscience) on a TOptical 96 real-time PCR thermal cycler (Biometra). All samples were run in triplicate. Data were calculated as fold change relative to *Gapdh* from the resulting threshold cycle number. Primers used are listed in Supplemental Table 3.

### Statistics.

Statistical analyses were performed using Prism 6 (GraphPad Prism). Two-way ANOVA followed by Bonferroni’s post hoc comparisons were used to analyze airway resistance data. One-way ANOVA followed by Bonferroni’s post hoc comparisons were performed to determine statistical significance between multiple groups, whereas unpaired Student’s 2-tailed *t* test was used to evaluate differences between 2 groups. *P* values less than 0.05 were considered to be significant.

### Study approval.

All animal studies were approved by Academia Sinica IACUC, Taiwan, and all experiments were performed according to the guidelines of the IACUC.

## Author contributions

YJC initiated and designed the study. CLPT, ACYL, JCW, PYC, YLC, and YTT conducted experiments and analyzed the data. CLPT and ACYL wrote the manuscript. SYC and YJC reviewed and edited the manuscript. Authorship order for co–first authors was determined via mutual agreement between CLPT and ACYL.

## Supplementary Material

Supplemental data

## Version 1 12/08/2022

Electronic publication
