# Supplementary material for: Identification of a PD-L1+Tim-1+ iNKT subset that protects against fine particulate matter–induced airway inflammation
Source: JCI Insight. 2022 Dec 8;7(23):e164157. doi: 10.1172/jci.insight.164157 (PMC9746902; doi:10.1172/jci.insight.164157)
Supplement: Supplemental data [file jciinsight-7-164157-s018.pdf]

ONLINE SUPPLEMENTARY MATERIAL

Identification of a PD-L1$^+$ Tim-1$^+$ iNKT subset that protects against fine particulate matter-induced airway inflammation

Christina Li-Ping Thio,[1†] Alan Chuan-Ying Lai,[1†] Jo-Chiao Wang,[1] Po-Yu Chi,[1] Ya-Lin Chang,[1] Yu-Tse Ting,[1] Shih-Yu Chen,[1] and Ya-Jen Chang,[1,2*]

Affiliations:

[1]Institute of Biomedical Sciences, Academia Sinica, Taipei, Taiwan.

[2]Institute of Translational Medicine and New Drug Development, China Medical University, Taichung, Taiwan.

[†] These authors are co-first authors.

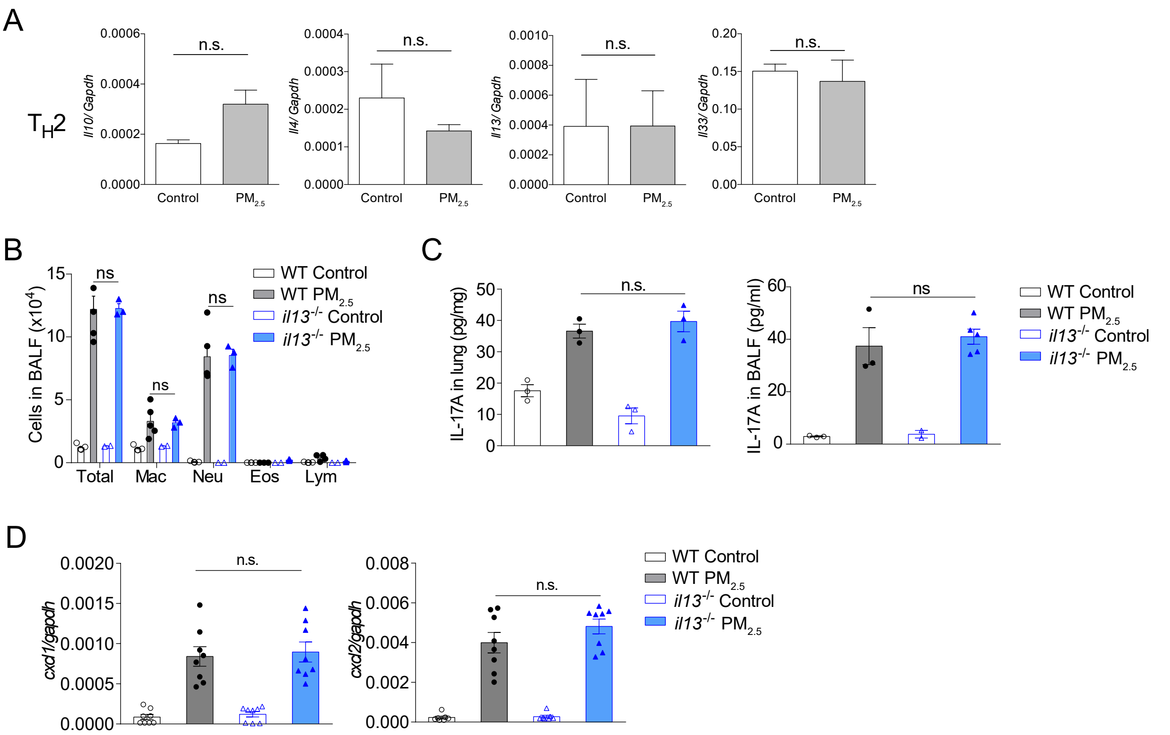

Supplemental Figure 1. $PM_{2.5}$-induced inflammation is independent of T_H2 response. BALB/c (WT) (**A-D**) and *Il13*$^{-/-}$ (**B-D**) mice received daily intranasal exposure of $PM_{2.5}$ for three days and sacrificed one day after the last exposure. (**A**) Transcript expression levels of T_H2-associated cytokines (*Il10*, *Il4*, *Il13*, and *Il33*) in the lungs. (**B**) Cellular composition (Mac, macrophage; Neu, neutrophil; Eos, eosinophil; Lym, lymphocyte) in BALF. (**C**) Levels of IL-17A in the lungs and BALF. (**D**) Transcript expression levels of *Cxcl1* and *Cxcl2* in the lungs. Data are expressed as mean ± SEM from 2 independent experiments (n=2-8 per group). Statistical analysis was performed using an unpaired two-tailed *t* test (**A**) or one-way ANOVA (**B-D**). n.s., Not significant.

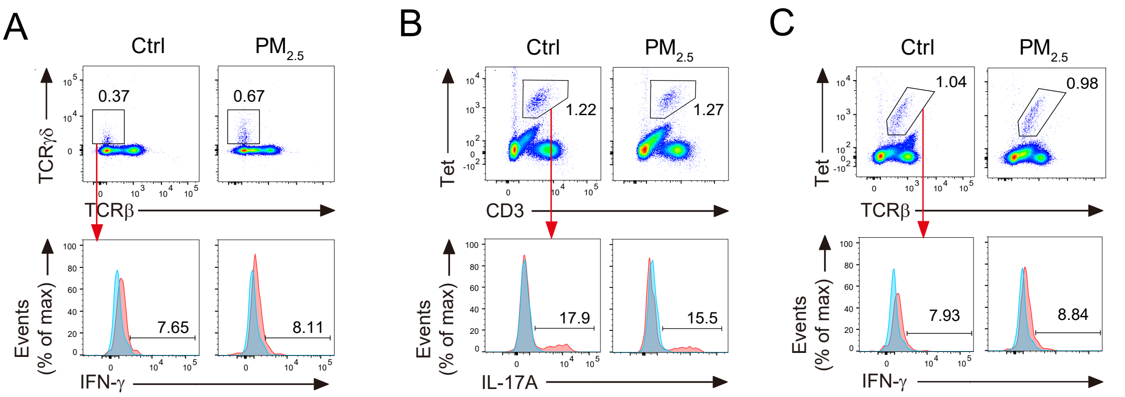

Supplemental Figure 2. IL-17A and IFN-γ production by γδ T and iNKT cells. BALB/c received daily intranasal exposure of PM2.5 for three days and sacrificed one day after the last exposure. (**A**) Representative flow cytometry plot showing IFN-γ production in γδ T cells (TCRγδ^{+} TCRβ^{-} cells). (**B-C**) Representative flow cytometry plot showing IL-17A (**B**) and IFN-γ (**C**) production in iNKT cells (CD1d-tetramer^{+} CD3^{+} TCRβ^{+} cells).

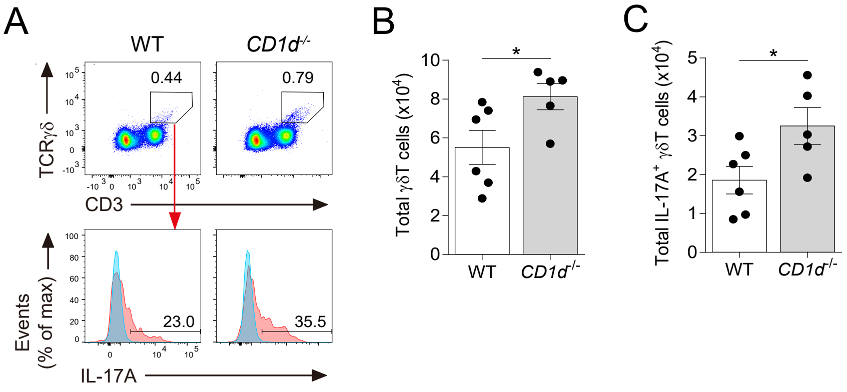

Supplemental Figure 3. IL-17A production by γδ T cells in naïve WT and *CD1d*$^{-/-}$ mice. BALB/c (WT) and *CD1d*$^{-/-}$ mice received daily intranasal exposure of $PM_{2.5}$ for three days and sacrificed one day after the last exposure. (**A**) Representative flow cytometry plot showing IL-17A^{+} γδ T cells (CD3^{+} TCRγδ^{+} cells). (**B**) Total γδ T cells and (**C**) IL-17A^{+} γδ T cells in naïve WT and *CD1d*$^{-/-}$ mice, assessed as in **A**. Data are expressed as mean ± SEM from 2 independent experiments (n=5-6 per group). Statistical analysis was performed using an unpaired two-tailed *t* test. **P*<.05.

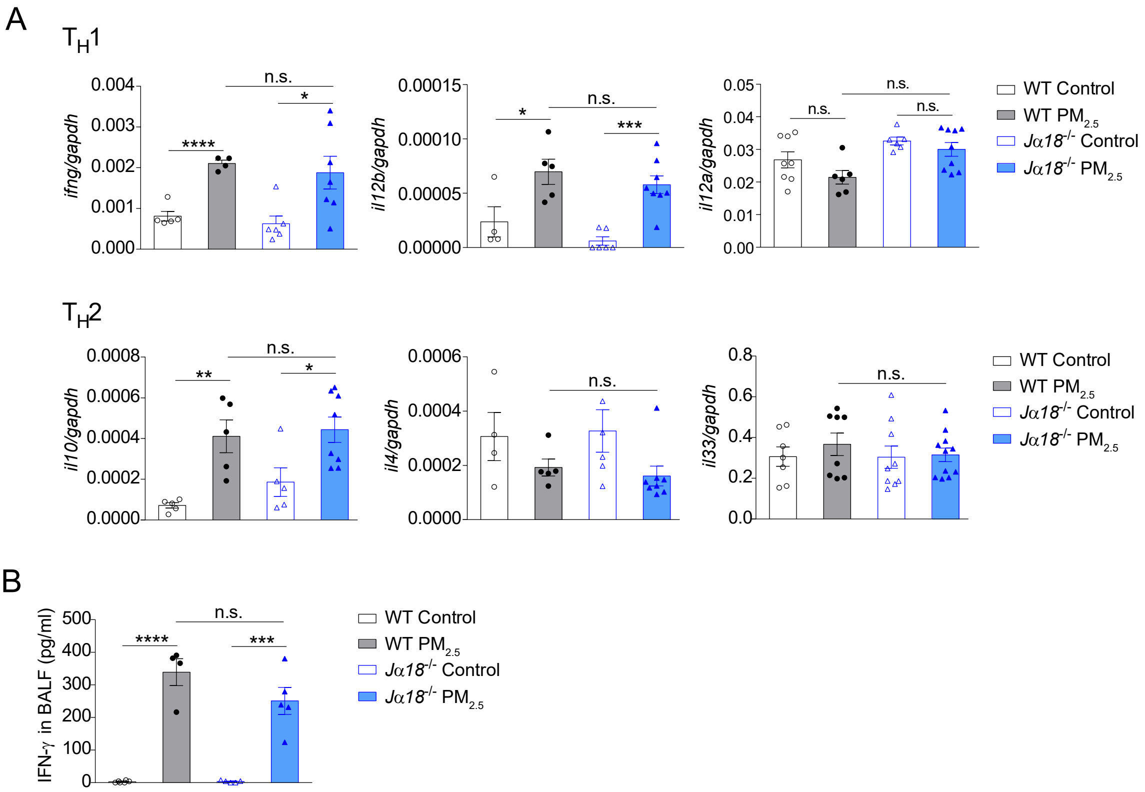

Supplemental Figure 4. iNKT cell-deficiency does not alter T_H1 and T_H2 responses after $PM_{2.5}$ treatment. BALB/c and *Jα18*$^{-/-}$ mice received daily intranasal exposure of $PM_{2.5}$ for three days and sacrificed one day after the last exposure. (**A**) Levels of T_H1 (*Ifng*, *Il12a*, and *Il12b*) and T_H2 (*Il10*, *Il4*, and *Il33*) mRNA in the lungs of mice. (**B**) Level of IFN-γ in the BALF. Data are expressed as mean ± SEM from 2 independent experiments (n=4-8 per group). Statistical analysis was performed using one-way ANOVA. **P*<.05, ***P*<.01, ****P*<.001, and **** *P*<.0001. n.s., Not significant.

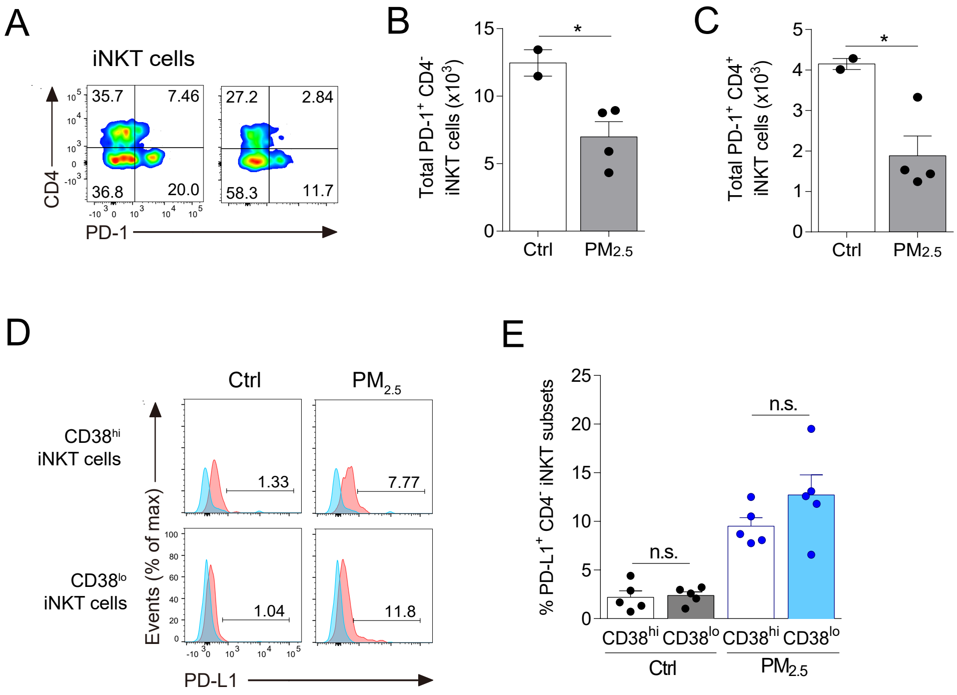

Supplemental Figure 5. PD-1 and PD-L1 expression on iNKT cells. BALB/c received daily intranasal exposure of PM2.5 for three days and sacrificed one day after the last exposure. (**A**) Representative flow cytometry plot showing PD-1 expression on the $CD4^+$ and $CD4^-$ iNKT cell subsets (Gated from CD1d-tetramer$^+$ TCRβ^+ cells). (**B**) Total PD-1$^+$ $CD4^-$ iNKT cells and (**C**) PD-1$^+$ $CD4^+$ iNKT cells, assessed as in **A**. (**D**) Representative histogram showing PD-L1 expression on $CD38^{hi}$ and $CD38^{lo}$ iNKT cell subsets (Gated from CD1d-tetramer$^+$ TCRβ^+ $CD4^-$ cells). Blue solid line: Isotype-matched control; Red solid line: Antibody staining. (**E**) Frequencies of PD-L1-expressing $CD38^{hi}$ and $CD38^{lo}$ $CD4^-$ iNKT subsets, assessed as in **D**. Data are representative of one experiment (n=2-5 per group). Statistical analysis was performed using an unpaired two-tailed *t* test (**B, C**) or one-way ANOVA (**E**). *$P<.05$. n.s., Not significant.

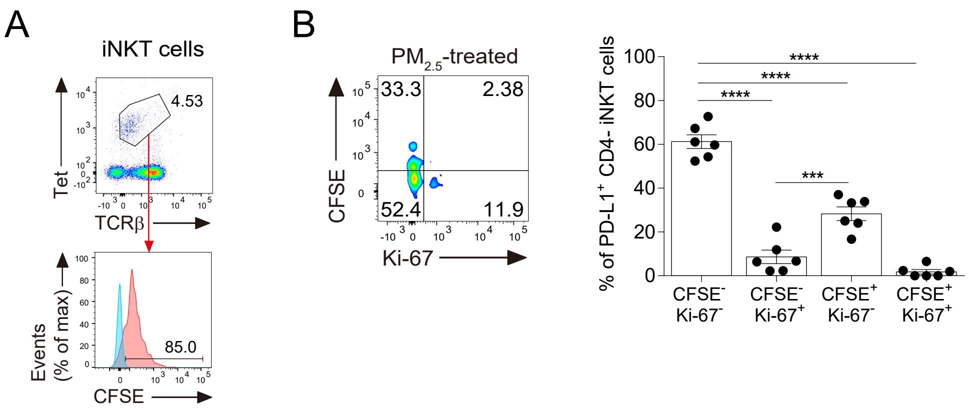

Supplemental Figure 6. Expression of CFSE and Ki-67 in PD-L1$^+$ CD4$^-$ iNKT cells. BALB/c mice received daily intranasal exposure of $PM_{2.5}$ for three days and sacrificed one day after the last exposure. CFSE (2 µg/g mouse) was administered intravenously after the second exposure to $PM_{2.5}$. (**A**) Representative flow cytometry plot showing CFSE$^+$ iNKT cells in the blood 48 hours after CFSE labeling. (**B**) Representative flow cytometry plot and quantitative data showing CFSE- and Ki-67-expressing PD-L1$^+$ CD4$^-$ iNKT cell subset, gated as in **Figure 6E**. Data are shown as mean ± SEM from 2 independent experiments (n=6 per group). Statistical analysis was performed using one-way ANOVA (**B**). *** P<.001 and **** P<.0001.

Supplemental tables

Supplemental Table 1: List of antibodies used for CyTOF

Antigen panel	Symbol	Mass	Antibody clone	Brand
CD90	In	113	30-H12	Biolegend
TCRβ	In	115	H57-597	Biolegend
CD44	Cd	116	IM7	BD Biosciences
CD11b	Ce	140	M1/70	Biolegend
CD69	Pr	141	H1.2F3	Biolegend
CD45	Nd	142	30-F11	Biolegend
CD11c	Nd	143	HL3	BD Biosciences
Gr1	Nd	144	RB6-8C5	Biolegend
CD4	Nd	145	RM4-5	Fluidigm
CD3	Sm	147	17A2	Biolegend
CD64	Nd	148	X54-5/7.1	Biolegend
CD19	Sm	149	6D5	Fluidigm
CD27	Nd	150	LG.3A10	Fluidigm
Ly6C	Eu	151	HK1.4	Biolegend
Ki-67	Sm	152	SolA15	eBioscience
CD8a	Gd	155	53-6.7	Biolegend
CD1d tetramer	Gd	156		NIH
Foxp3	Gd	158	FJK-16S	eBioscience
PD-1	Tb	159	29F.1A12	Biolegend
GATA3	Gd	160	TWAJ	eBioscience
Tbet	Dy	161	O4-46	Fluidigm
TCRγδ	Dy	162	GL3	Biolegend
CD62L	Dy	164	MEL-14	Fluidigm
NK1.1	Ho	165	PK136	Fluidigm
cKit	Er	166	2B8	Biolegend
NKp46	Er	167	29A1.4	Biolegend
RORγt	Er	168	600214	Fluidigm
F4/80	Tm	169	BM8	Biolegend
CD137 (41BB)	Er	170	17B5	Biolegend
CD86	Yb	172	GL-1	Biolegend
FceRI	Yb	173	MAR-I	Biolegend
mSiglecF	Yb	174	E50-2440	BD Biosciences
CD127	Lu	175	A7R34	Fluidigm
ST2	Yb	176	DIH9	Biolegend
MHCII	Bi	209	M5/114.15.2	Fluidigm

DNA	Ir	191/193		
Cisplatin Viability	Pt	195		

Supplemental Table 2: Gating strategy for identifying $CD45^+$ $CD90^+$ lymphocyte subsets by CyTOF analysis

Immune cell subsets	Gating strategy
CD4 T cells	$CD3^+$ $TCR\beta^+$ $CD4^+$ $CD8^-$
CD8 T cells	$CD3^+$ $TCR\beta^+$ $CD8^+$ $CD4^-$
$CD44^+$ γδ T cells	$CD3^+$ $TCR\beta^-$ $TCR\gamma\delta^+$ $CD44^+$
$CD27^+$ γδ T cells	$CD3^+$ $TCR\beta^-$ $TCR\gamma\delta^+$ $CD27^+$
NKT cells	$CD3^+$ $TCR\beta^+$ CD1d-tetramer$^+$
Treg	$CD3^+$ $TCR\beta^+$ $Foxp3^+$
NK cells	$CD3^-$ $NK1.1^+$ $NKp46^+$ $Tbet^+$
ILC2	$CD3^-$ $TCR\beta^-$ $CD127^+$ $ROR\gamma t^-$ $Tbet^-$ $GATA3^+$
ILC3	$CD3^-$ $TCR\beta^-$ $CD127^+$ $GATA3^-$ $Tbet^-$ $ROR\gamma t^+$

*NKT cells: Natural killer T cells; Treg: Regulatory T cells; NK cells: Natural killer cells; ILC2: Group 2 innate lymphoid cells; ILC3: Group 3 innate lymphoid cells

Supplemental Table 3: Lists of primers used

Gene	Forward	Reverse
Il17a	TCCAGAAGGCCCTCAGACTA	ACACCCACCAGCATCTTCTC
Ifng	GGCCA TCAGC AACAA CATAA GCGT	TGGGT TGTTG ACCTC AAACT TGGC
Il12a	CTGTGCCTTGGTAGCATCTATG	GCAGAGTCTCGCCATTATGATTC
Il12b	GTCCTCAGAAGCTAACCATCTCC	CCAGAGCCTATGACTCCATGTC
Il33	ATTTC CCCGG CAAAG TTCAG	AACGG AGTCT CATGC AGTAG A
Il10	GGTTGCCAAGCCTTATCGGA	ACCTGCTCCACTGCCTTGCT
Il4	GGTCTCAACCCCCAGCTAGT	GCCGATGATCTCTCTCAAGTGAT
Il13	CCTGGCTCTTGCTTGCCTT	GGTCTTGTGTGATGTTGCTCA
Il1b	GAAAT GCCAC CTTTT GACAG TG	CTGGA TGCTC TCATC AGGAC A
Il23a	ATGCT GGATT GCAGA GCAGT A	ACGGG GCACA TTATT TTTAG TCT
Cxcl1	AAAAGGTGTCCCCAAGTA	AAGCAGAACTGAACTACCATCG
Cxcl2	GGGAGAGGGTGAGTTGGG	GCACACTCCTTCCATGAAAGC
